# Hungry bone syndrome in peritoneal dialysis patients after parathyroid surgery

**DOI:** 10.1530/EC-23-0107

**Published:** 2023-09-22

**Authors:** Heng Yeh, Hsuan Yeh, Chun-Cheng Chiang, Ju-Ching Yen, I-Kuan Wang, Shou-Hsuan Liu, Cheng-Chia Lee, Cheng-Hao Weng, Wen-Hung Huang, Ching-Wei Hsu, Tzung-Hai Yen

**Affiliations:** 1 Department of Emergency Medicine, Chang Gung Memorial Hospital, Linkou, Taoyuan, Taiwan; 2 College of Medicine, Chang Gung University, Taoyuan, Taiwan; 3 Department of Nephrology, Clinical Poison Center, Chang Gung Memorial Hospital, Linkou, Taoyuan, Taiwan; 4 Department of Gastroenterology and Hepatology, Chang Gung Memorial Hospital, Linkou, Taoyuan, Taiwan; 5 College of Medicine, China Medical University, Taichung, Taiwan; 6 Department of Nephrology, China Medical University Hospital, Taichung, Taiwan

**Keywords:** hungry bone syndrome, parathyroidectomy, peritoneal dialysis, secondary hyperparathyroidism, hypocalcemia

## Abstract

Secondary hyperparathyroidism (SHPT) is a common complication of end-stage kidney disease (ESKD). Hungry bone syndrome (HBS) occurs frequently in patients on maintenance dialysis receiving parathyroidectomy for refractory SHPT. However, there is scanty study investigating the clinical risk factors that predict postoperative HBS, and its outcome in peritoneal dialysis (PD) patients. We conducted a single-center retrospective study to analyze 66 PD patients who had undergone parathyroidectomy for secondary hyperparathyroidism at Chang Gung Memorial Hospital between 2009 and 2019. The patients were stratified into two groups based on the presence (*n*=47) or absence (*n*=19) of HBS after parathyroidectomy. Subtotal parathyroidectomy was the most common surgery performed (74.2%), followed by total parathyroidectomy with autoimplantation (25.8%). Pathological examination of all surgical specimens revealed parathyroid hyperplasia (100%). Patients with HBS had lower levels of postoperative nadir corrected calcium, higher alkaline phosphate (ALP), and higher potassium levels compared with patients without HBS (all *P*<0.05). A multivariate logistic regression model confirmed that lower preoperative serum calcium level (OR 0.354, 95% CI 0.133–0.940, *P*=0.037), higher ALP (OR 1.026, 95% CI 1.008–1.044, *P*=0.004), and higher potassium level (OR 6.894, 95% CI 1.806–26.317, *P*=0.005) were associated with HBS after parathyroidectomy. Patients were followed for 58.2±30.8 months after the surgery. There was no significant difference between HBS and non-HBS groups in persistence (*P*=0.496) or recurrence (*P*=1.000) of hyperparathyroidism. The overall mortality rate was 10.6% with no significant difference found between both groups (*P*=0.099). We concluded that HBS is a common complication (71.2%) of parathyroidectomy for SHPT and should be managed appropriately.

## Introduction

Secondary hyperparathyroidism (SHPT) is a common complication of chronic kidney disease (CKD) characterized by the hypersecretion of parathyroid hormone (PTH) and the parathyroid gland hyperplasia stimulated by CKD-related chronic hyperphosphatemia, hypocalcemia, and 1,25-dihydroxyvitamin D (1,25(OH)2D) deficiency (1), affecting nearly 90% of those with end-stage kidney disease (ESKD) receiving chronic renal replacement therapy (RRT) (2). SHPT is associated with increased risks of fracture, vascular calcification, coronary atherosclerotic disease, and anemia resistant to erythropoietin therapy, leading to substantial morbidity and mortality in patients on dialysis (1, 3). The initial management of SHPT of renal origin is optimizing serum calcium and phosphorus levels with low-phosphorus diet along with medications including phosphate binders, vitamin D derivatives, and calcimimetics (4), while patients nonresponsive to maximal medical treatments are considered as refractory SHPT and require surgical intervention. Persistent elevated serum intact parathyroid hormone (iPTH) level higher than 800 pg/mL associated with hypercalcemia and/or hyperphosphatemia refractory to medical therapy is generally accepted as an indication for parathyroidectomy (5). Surgical methods employed in SHPT are total parathyroidectomy, subtotal parathyroidectomy, and total parathyroidectomy with autotransplantation. Among them, total parathyroidectomy has fallen out of favor because of the risk of permanent hypocalcemia, while subtotal parathyroidectomy and total parathyroidectomy with autotransplantation are more commonly applied to patients in the contemporary practice (6). Despite optimization of surgical approach, postoperative hypocalcemia due to unopposed osteoblast uptake of minerals after acute decrease in PTH levels remains a common complication after parathyroidectomy in these patients and can lead to poor outcomes.

Severe hypocalcemia after parathyroidectomy, also known as hungry bone syndrome (HBS), is defined as a drop in serum calcium concentration to less than 8.4 mg/dl and/or prolonged hypocalcemia for more than four days post operation (7). HBS often presents with mild clinical symptoms including weakness, paresthesia, tingling in the extremities, positive Chvostek or Trousseau sign, and muscle cramps, but may also develop life-threatening sequelae such as seizures, laryngeal stridor, cardiac arrythmia, even mortality in rare cases (8). To say the least, HBS was associated with longer hospital stay or readmission (8). Among patients with ESKD on long-term dialysis, those receiving peritoneal dialysis (PD) treatment are reported to be positively associated with younger age, higher educational level, and higher scores of activities of daily living (9). Therefore, judicious preoperative evaluation, postoperative surveillance, and accurate prediction of the occurrence and the outcome of HBS in this patient group are particularly critical from the societal perspective. Although multiple studies have identified several patient factors and perioperative biochemical parameters to estimate the risk of postparathyroidectomy hypocalcemia and HBS (10, 11, 12, 13), few studies have focused on the risk factors of HBS after parathyroidectomy in PD patients. This study aimed to examine the clinical and laboratory data to identify risk factors of HBS after parathyroidectomy for SHPT in PD population to prevent the occurrence of HBS and to optimize clinical management.

## Materials and methods

### Ethical statement

The study was approved by the Institutional Board Review of the Chang Gung Medical Foundation Institutional Review Board (numbered 202000663B0). Because this study involved retrospective review of existing data, Institutional Review Board approval was obtained, but without specific written informed consent from the patients. All methods were performed in accordance with the guidelines and regulations of the Medical Ethics Committee of Chang Gung Memorial Hospital, as well as with the Declaration of Helsinki.

### Patients

Between 2009 and 2019, 66 PD patients underwent parathyroidectomy for secondary hyperparathyroidism at Chang Gung Memorial Hospital. Demographic, clinical, and biochemical data were obtained from electronic medical records. Age, gender, body mass index, etiology of ESKD, mode and duration of RRT, smoking and drinking status, past medical history, surgical history, medications, indication for parathyroidectomy, surgical procedure and pathological report, postoperative symptoms and hospital stay, lifespan within the study period, were retrieved. Preoperative and postoperative biochemical parameters, including serum calcium, serum phosphate, serum sodium, serum potassium, serum chloride, serum albumin, serum ALP, and serum iPTH, were also collected. All biochemical parameters were assessed using automatic and standardized procedures at the central laboratory of Chang Gung Memorial Hospital.

### Patient groups

Patients who met the inclusion criteria were divided into two groups according to the presence (*n* = 47) or absence (*n* = 19) of HBS after parathyroidectomy.

### Inclusion and exclusion criteria

**Figure 1:**
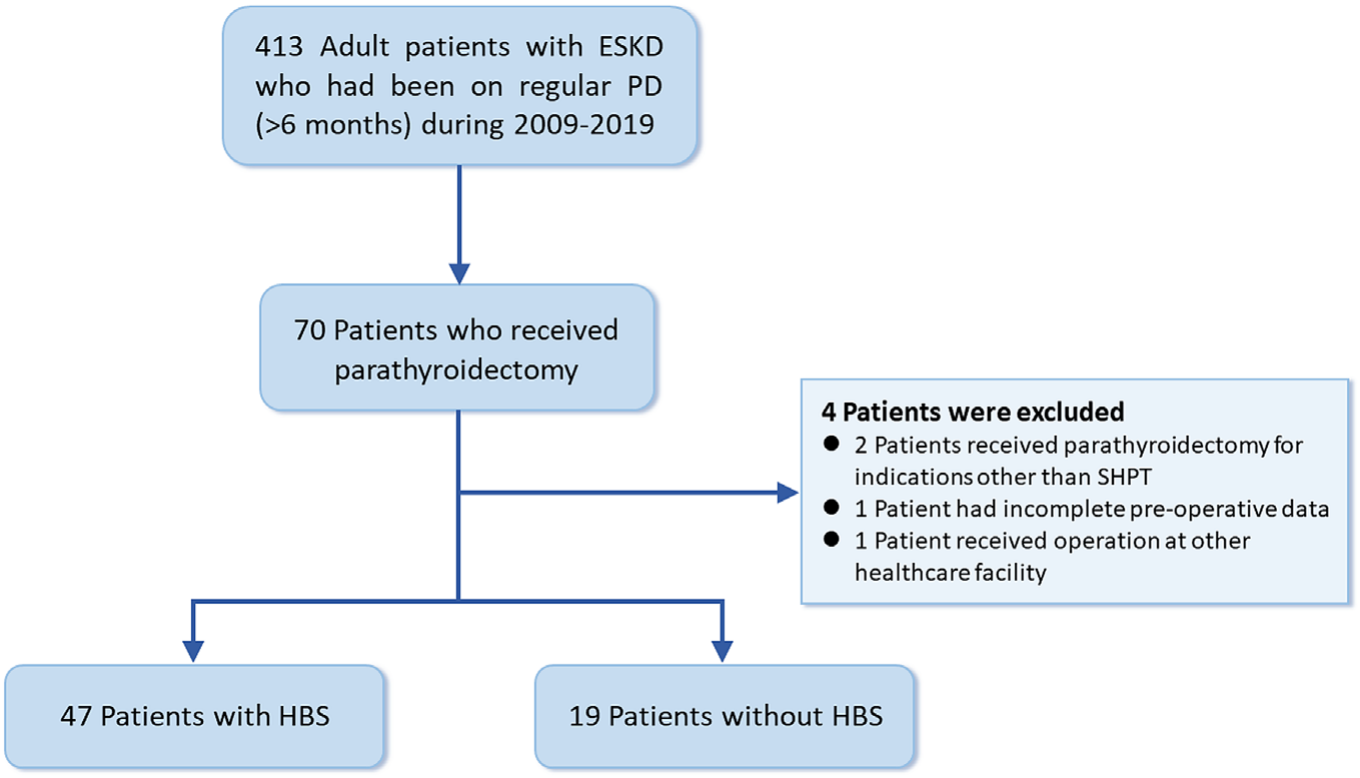
Flowchart showing the enrolment and status of patients. ESKD, end-stage kidney disease; HBS, hungry bone syndrome; PD, peritoneal dialysis; SHPT, secondary hyperparathyroidism.

Inclusion criteria were adult patients aged 18 and above with SHPT who underwent chronic PD and had parathyroidectomy for the first time during 2009–2019 (Fig. 1). Patients who had been receiving PD for less than 6 months, those who had received parathyroidectomy at other healthcare facilities, those who received parathyroidectomy for indications other than SPHT, and those who had incomplete preoperative data were excluded from the study.

### Definitions

HBS was defined as serum corrected calcium lower than 8.4 mg/dL that lasted more than 3 days. The normal range for intact parathyroid hormone is 15–65 pg/mL. In the present study, persistent hyperparathyroidism was defined as failure of iPTH levels to fall below the upper limit of normal (>65 pg/mL) within 6 months of parathyroidectomy. Previous studies used variable cutoffs in defining recurrent SHPT. Some studies used cutoffs ranging from 200 to 235 pg/mL (14, 15, 16), whereas other study simply used the upper limit of normal for the PTH assay (65 pg/mL) (17). Using the upper limit of normal for the PTH assay as the threshold is extremely stringent but not realistic and may not yield clinically significant information (6). Therefore, we adopted 200pg/mL as the cutoff and defined recurrent hyperparathyroidism as elevated levels of iPTH (>200 pg/mL) that developed more than 6 months after successful parathyroidectomy.

### PD regimen

Dialysate/plasma creatinine ratio, peritoneal transport characteristics, weekly creatinine clearance and weekly Kt/V_urea_ were surveyed by standardized methods. PD prescriptions were based on the peritoneal membrane characteristics determined by the peritoneal equilibration test. Intermittent therapy was prescribed to patients with high membrane transport and continuous therapy to those with average or low membrane transport. Icodextrin-based (7.5 g/dL) or Dianeal PD-2 (Baxter Healthcare, Deerfield, IL, USA) solution containing 1.5%, 2.5%, or 4.25% dextrose with low (2.5 mEq/L) or standard (3.5 mEq/L) calcium concentrations were used according to the patients’ peritoneal transport characteristics and serum calcium levels to maintain adequate ultrafiltration and normal calcium levels. Dialysis prescription aimed at obtaining a total Kt/V of at least 1.8 per week.

### Prevention and treatment for HBS

All patients undergoing parathyroidectomy were started on calcitriol 0.25 µg twice daily for one to two days before surgery if they had not been on any form of vitamin D treatment. After surgery, all patients, no matter which type of dialysates they received preoperatively, were transitioned to a normal calcium (3.5 mEq/L) dialysis bath. All patients were given 1 to 2pc oral calcium carbonate 500 mg or calcium acetate 667 mg twice or thrice daily. Serum calcium levels were monitored every 6–8 h starting from the time immediately after surgery. If serum calcium level was below 7.5 mg/dL, extra 10 mL of 10% calcium chloride solution (272 mg elemental calcium) was given intravenously. The frequency of serum calcium level monitoring and the dosage of medication were adjusted by the attending physician during the hospitalization. Patients were discharged when calcium levels remained stable in the normal range and received regular follow-up and medication adjustment at PD outpatient department.

### Statistical analysis

For comparison between the two groups, Student’s *t*-test was used for quantitative variables, whereas the chi-squared or Fisher’s exact test was used for categorical variables. For normally distributed continuous variables, means and 95% confidence interval (CI) are presented unless indicated otherwise. For non-normally distributed continuous variables, geometric means and 95% CI are reported. Univariate binary logistic regression analysis was performed to analyze potential variables (all variables from Table 2 to 4) that may be associated with the development of HBS and was reported as odds ratio (OR) and 95% CI. Stepwise backward multivariate binary logistic regression was then performed to control for confounders and to analyze variables that were found to be significant (*P* < 0.05) in the univariate analysis. The analyses were performed using SPSS version 25.0 for Mac (IBM).

## Results

Table 1 presents the baseline characteristics of patients with parathyroidectomy, stratified by the presence (47 of 66 or 71.2%) or absence (19 of 66 or 28.8%) of HBS. At the time of surgery, the patients were 49.4 ± 11.9 years old and had been receiving peritoneal dialysis for 86.8 ± 37.7 months. Patients with HBS tended to have shorter duration of dialysis compared to those without HBS, although the difference failed to reach statistical significance (81.1 ± 38.7 vs 101.0 ± 31.8 months, *P* = 0.051). Hypertension and cardiovascular diseases were present in 92.4% and 21.2% of the patients, respectively. Hypertension (42.4%), glomerulonephritis (36.4%), and diabetes (9.1%) were the three leading known causes of ESKD. In the HBS group, two patients had undergone hemodialysis (HD) and one patient had undergone an allograft renal transplant before transitioning to regular PD, whereas none of the patients in the non-HBS group had a prior history of renal replacement therapy apart from PD. No significant differences in baseline variables were noted between both groups (*P* > 0.05).

**Table 1:** Baseline characteristics of peritoneal dialysis patients underwent parathyroidectomy, stratified by the presence or absence of hungry bone syndrome (*n* = 66).

Variable	All patients (*n* = 66)	Patients with hungry bone syndrome (*n* = 47)	Patients without hungry bone syndrome (*n* = 19)	*P*
Age, year	49.4 ± 11.9	48.6 ± 12.2	51.4 ± 10.9	0.382
Female gender,* n* (%)	51 (77.3)	38 (80.9)	13 (68.4)	0.335
Body mass index, kg/m^2^	24.0 ± 3.4	24.3 ± 3.8	23.2 ± 2.1	0.220
Duration of dialysis, month	86.8 ± 37.7	81.1 ± 38.7	101.0 ± 31.8	0.051
Hypertension, *n* (%)	61 (92.4)	42 (89.4)	19 (100)	0.311
Cardiovascular disease, *n* (%)	14 (21.2)	11 (23.4)	3 (15.8)	0.741
Hepatitis B virus carrier, *n* (%)	10 (15.2)	6 (12.8)	4 (21.1)	0.456
Hepatitis C virus carrier, *n* (%)	2 (3.0)	2 (4.3)	0 (0.0)	1.000
Smoking habit, *n* (%)	6 (9.1)	4 (8.5)	2 (10.5)	1.000
Etiology of end-stage kidney disease				0.697
Diabetes, *n* (%)	6 (9.1)	5 (10.6)	1 (5.3)	
Hypertension, *n* (%)	28 (42.4)	18 (38.3)	10 (52.6)	
Glomerulonephritis, *n* (%)	24 (36.4)	17 (36.2)	7 (36.8)	
Polycystic kidney disease, *n* (%)	1 (1.5)	1 (2.1)	0 (0.0)	
Unknown, *n* (%)	7 (10.6)	6 (12.8)	1 (5.3)	
History of renal replacement therapy other than peritoneal dialysis				
Hemodialysis, *n* (%)	1 (1.5)	1 (2.1)	0 (0.0)	0.522
Transplant, *n* (%)	2 (3.0)	2 (4.3)	0 (0.0)	0.361

As shown in Table 2, patients who received PD had high blood levels of iPTH (1768.4 ± 1480.3 pg/mL), corrected calcium (10.6 ± 0.9 mg/dL) and phosphate (6.3 ± 1.3 mg/dL) before surgery. Uric acid (7.1 ± 1.2 mg/dL) and triglyceride (214.8 ± 167.4 mg/dL) levels were also higher. In addition, preoperative blood tests revealed that patients with HBS had higher levels of potassium (4.0 ± 0.6 vs 3.6 ± 0.6 mEq/L, *P* = 0.015) and alkaline phosphatase (ALP) (178.2 ± 87.5 vs 115.0 ± 29.6 U/L, *P* = 0.003), and lower corrected calcium (10.4 ± 0.9 vs 11.0 ± 0.8 mg/dL, *P* = 0.014) than those without HBS. No significant differences in other laboratory variables were noted. Patients received adequate doses of dialysis and had optimal nutritional status as shown by the average Kt/*V* (2.3 ± 0.3) and normalized protein catabolic rate (1.1 ± 0.2), respectively. No significant difference in dialysate calcium concentration and PD modality were observed between the two groups. Nonetheless, patients in the HBS group had significantly greater residual urine volume (167.8 ± 257.3 vs 57.4 ± 105.0 mL, *P* = 0.015).

**Table 2:** Preoperative laboratory and dialysis-related data of peritoneal dialysis patients underwent parathyroidectomy, stratified by the presence or absence of hungry bone syndrome (*n* = 66).

Variable	All patients (*n* = 66)	Patients with hungry bone syndrome (*n* = 47)	Patients without hungry bone syndrome (*n* = 19)	*P*
Hemogram				
White blood cell count, 10^3^/µL	7.6 ± 2.7	7.4 ± 2.8	8.1 ± 2.7	0.403
Red blood cell count, 10^6^/µL	3.3 ± 0.6	3.3 ± 0.6	3.3 ± 0.7	0.944
Hemoglobin, g/dL	9.5 ± 1.7	9.5 ± 1.7	9.7 ± 1.8	0.689
Hematocrit, %	28.8 ± 5.1	28.8 ± 5.0	28.9 ± 5.4	0.936
Mean corpuscular volume, fL	87.4 ± 6.2	87.3 ± 6.5	87.6 ± 5.4	0.884
Platelet count, 10^3^/µL	290.6 ± 89.8	288.4 ± 91.3	296.0 ± 88.2	0.758
Biochemistry				
Intact parathyroid hormone, pg/mL	1768.4 ± 1480.3	1951.4 ± 1708.1	1315.9 ± 390.7	0.115
Aspartate aminotransferase, U/L	20.4 ± 9.3	19.1 ± 8.6	23.5 ± 10.4	0.082
Alanine aminotransferase, U/L	18.7 ± 21.1	19.0 ± 24.5	17.8 ± 7.7	0.829
Total bilirubin, mg/dL	0.3 ± 0.1	0.3 ± 0.1	0.3 ± 0.2	0.994
Fasting glucose, mg/dL	113.0 ± 51.2	112.9 ± 58.2	113.5 ± 29.5	0.966
Uric acid, mg/dL	7.1 ± 1.2	7.1 ± 1.2	7.1 ± 1.4	0.862
Blood urea nitrogen, mg/dL	69.1 ± 18.3	70.5 ± 18.8	65.7 ± 16.8	0.338
Creatinine, mg/dL	12.2 ± 2.6	12.2 ± 2.5	12.3 ± 2.9	0.822
Potassium, mEq/L	3.9 ± 0.6	4.0 ± 0.6	3.6 ± 0.6	0.015^a^
Sodium, mEq/L	135.3 ± 3.7	135.2 ± 3.6	135.6 ± 4.2	0.726
Corrected calcium, mg/dL	10.6 ± 0.9	10.4 ± 0.9	11.0 ± 0.8	0.014^a^
Phosphate, mg/dL	6.3 ± 1.3	6.4 ± 1.3	6.2 ± 1.5	0.687
Albumin, g/dL	3.7 ± 0.4	3.7 ± 0.4	3.7 ± 0.4	0.731
Alkaline phosphatase, U/L	159.7 ± 80.4	178.2 ± 87.5	115.0 ± 29.6	0.003^b^
Total cholesterol, mg/dL	197.4 ± 38.3	202.5 ± 39.5	184.8 ± 32.9	0.091
Triglycerides, mg/dL	214.8 ± 167.4	201.4 ± 147.2	247.2 ± 209.5	0.320
Iron, µg/dL	64.9 ± 25.4	64.7 ± 23.1	65.5 ± 31.2	0.912
Total iron binding capacity, µg/dL	297.1 ± 60.1	287.8 ± 51.5	320.4 ± 74.2	0.051
Transferrin saturation, %	22.1 ± 8.5	22.9 ± 8.7	20.3 ± 7.7	0.287
Ferritin, ng/mL	301.6 ± 357.3	316.1 ± 367.5	268.0 ± 339.6	0.628
Dialysis-related data Dialysate calcium concentration				0.979
2.5 mEq/L, n (%)	45 (68.2)	32 (68.1)	13 (68.4)	
3.5 mEq/L, n (%)	21 (31.8)	15 (31.9)	6 (31.6)	
PD modality				0.133
CAPD, n (%)	49 (74.2)	32 (68.1)	17 (89.5)	
APD, n (%)	7 (10.6)	7 (14.9)	0 (0)	
CPAD/APD, n (%)	10 (15.2)	8 (17.0)	2 (10.5)	
Residual urine, mL/day	136.0 ± 225.8	167.8 ± 257.3	57.4 ± 105.0	0.015^a^
Kt/*V*_urea_	2.3 ± 0.3	2.3 ± 0.3	2.3 ± 0.3	0.962
Creatinine clearance, L/1.73 m^2^/week	56.9 ± 8.9	55.8 ± 7.7	59.8 ± 11.4	0.106
Normalized protein catabolic rate, g/kg/day	1.1 ± 0.2	1.1 ± 0.2	1.1 ± 0.3	0.896
Erythropoietin dose, unit/month	18872.7 ± 4615.0	18410.3 ± 5432.4	20000.0 ± 0.0	0.250

^a^*P* < 0.05, ^b^*P* < 0.01.

APD, automated peritoneal dialysis; CAPD, continuous ambulatory peritoneal dialysis; Kt/*V*_urea_, K dialyzer urea clearance; *t*, total dialysis session time; *V*, volume of distribution of urea.

As shown in Table 3, most patients underwent preoperative parathyroid ultrasound (81.8%) or parathyroid scan (60.6%). Calcium carbonate was the most frequently used phosphorus binder (95.5%), followed by aluminum hydroxide (8.3%), sevelamer (27.3%), and lanthanum carbonate (19.7%). Calcitriol and cinacalcet were used in 87.9% and 28.8% of the patients, respectively. No significant differences in preoperative examinations or medications were noted in both groups.

**Table 3:** Preoperative examinations and medications in peritoneal dialysis patients underwent parathyroidectomy, stratified by the presence or absence of hungry bone syndrome (*n* = 66).

Variable	All patients (*n* = 66)	Patients with hungry bone syndrome (*n* = 47)	Patients without hungry bone syndrome (*n* = 19)	*P*
Preoperative examinations				
Parathyroid ultrasound, *n* (%)	54 (81.8)	37 (78.7)	17 (89.5)	0.484
Parathyroid nuclear scan, *n* (%)	40 (60.6)	29 (61.7)	11 (57.9)	0.788
Preoperative medications				
Calcium carbonate, *n* (%)	63 (95.5)	45 (95.7)	18 (94.7)	1.000
Aluminum hydroxide, *n* (%)	12 (18.2)	8 (17.0)	4 (21.1)	0.732
Sevelamer, *n* (%)	18 (27.3)	14 (29.8)	4 (21.1)	0.554
Lanthanum carbonate,* n* (%)	13 (19.7)	9 (19.1)	4 (21.1)	1.000
Calcitriol, *n* (%)	58 (87.9)	41 (87.2)	17 (89.5)	1.000
Cinacalcet, *n* (%)	19 (28.8)	14 (29.8)	5 (26.3)	1.000

As shown in Table 4, subtotal parathyroidectomy was the most common surgery (74.2%) followed by total parathyroidectomy with autoimplantation (25.8%). Pathological examination confirmed parathyroid hyperplasia (100%). Further, 47 out of 66 (71.2%) patients developed HBS after parathyroidectomy. Compared with patients without HBS, patients with HBS had lower levels of nadir-corrected calcium (6.8 ± 0.7 vs 8.2 ± 0.7 mg/dL, *P* < 0.001) levels postoperatively.

**Table 4:** Outcome of peritoneal dialysis patients underwent parathyroidectomy, stratified by presence or absence of hungry bone syndrome (*n* = 66).

Variable	All patients (*n* = 66)	Patients with hungry bone syndrome (*n* = 47)	Patients without hungry bone syndrome (*n* = 19)	*P*
Type of surgery				0.759
Total parathyroidectomy with autoimplantation, *n* (%)	17 (25.8)	13 (27.7)	4 (21.1)	
Subtotal parathyroidectomy, *n* (%)	49 (74.2)	34 (72.3)	15 (78.9)	
Length of hospital stay, days	6.8 ± 5.0	7.2 ± 5.5	5.8 ± 3.1	0.308
Pathology finding				
Parathyroid gland hyperplasia, *n* (%)	66 (100.0)	47 (100.0)	19 (100.0)	1.000
Postsurgery blood tests				
Nadir corrected calcium, mg/dL	7.2 ± 1.0	6.8 ± 0.7	8.2 ± 0.7	<0.001^c^
Nadir intact parathyroid hormone, pg/mL	77.5 ± 222.9	50.0 ± 124.7	145.5 ± 364.4	0.116
Peak intact parathyroid hormone, pg/mL	526.6 ± 566.9	496.8 ± 556.5	600.0 ± 600.9	0.507
Parathyroidectomy-related complications				
Infection, *n* (%)	0 (0.0)	0 (0.0)	0 (0.0)	1.000
Hematoma, *n* (%)	0 (0.0)	0 (0.0)	0 (0.0)	1.000
Hoarseness, *n* (%)	2 (3.0)	2 (4.3)	0 (0.0)	1.000
Outcome of parathyroidectomy				
Persistent hyperparathyroidism, *n* (%)	13 (19.7)	8 (17.0)	5 (26.3)	0.496
Recurrence of hyperparathyroidism, *n* (%)	31 (47.0)	22 (46.8)	9 (47.4)	1.000
Second parathyroidectomy, *n* (%)	5 (7.6)	3 (6.4)	2 (10.5)	0.621
Postsurgery medications				
Calcium carbonate, *n* (%)	65 (98.5)	46 (97.9)	19 (100.0)	1.000
Aluminum hydroxide, *n* (%)	3 (4.5)	2 (4.3)	1 (5.3)	1.000
Sevelamer, *n* (%)	6 (9.1)	5 (10.6)	1 (5.3)	0.664
Lanthanum carbonate, *n* (%)	7 (10.6)	6 (12.8)	1 (5.3)	0.663
Calcitriol, *n* (%)	59 (89.4)	43 (91.5)	16 (84.2)	0.401
Cinacalcet, *n* (%)	4 (6.1)	4 (8.5)	0 (0.0)	0.316
Mortality	7 (10.6)	3 (6.4)	4 (21.1)	0.099
Follow-up duration, months	58.2 ± 30.8	56.3 ± 30.4	62.8 ± 32.1	0.703

^c^*P* < 0.001.

The mean hospitalization duration was 6.8 ± 5.0 days (Table 4), and no significant difference between both groups (7.2 ± 5.5 vs 5.8 ± 3.1 days, *P* = 0.308). Persistence and recurrence of hyperparathyroidism were observed in 13 (19.7%) and 31 (47.6%) patients, respectively, and 5 (7.6%) patients required second parathyroidectomy. No significant difference was found between both groups in the incidence of persistent (17.0% vs 26.3%, *P* = 0.496) or recurrent hyperparathyroidism (46.8% vs 47.4%, *P* = 1.000). Patients were followed up for 58.2 ± 30.8 months. The overall mortality rate was 10.6% with no significant difference found between HBS and non-HBS groups (6.4% vs 21.1%, *P* = 0.099) during the study period.

As shown in Table 5, the multivariate logistic regression model revealed that preoperative serum potassium and ALP levels were positive predictors of HBS (OR 6.894, 95% CI 1.806–26.317, *P* = 0.005; OR 1.026, 95% CI 1.008–1.044, *P* = 0.005), whereas preoperative corrected serum calcium level was a negative predictor of HBS (OR 0.354, 95% CI 0.133–0.940, *P* = 0.037).

**Table 5:** Analysis of risk factors for hungry bone syndrome using a logistic regression model (*n* = 66).

Variable	Univariate analysis			Multivariate analysis		
	Odds ratio	95% CI	*P*	Odds ratio	95% CI	*P*
Potassium (per 1 mEq/L increase)	3.235	1.196–8.751	0.021^a^	6.894	1.806–26.317	0.005^b^
Corrected calcium (per 1 mg/dL increase)	0.435	0.216–0.876	0.020^a^	0.354	0.133–0.940	0.037^a^
Alkaline phosphatase (per 1 U/L increase)	1.019	1.005–1.032	0.007^b^	1.026	1.008–1.044	0.004^b^

^a^*P* < 0.05, ^b^*P* < 0.01.

## Discussion

In this study, we conducted a single-center retrospective cohort analysis with longitudinal data collected at the Linkou Chang Gung Memorial Hospital, a tertiary referral center that had a capacity of 3700 beds and 100,000 annual admissions in Taiwan. The major findings of the present study were as follows: (i) The incidence of HBS after parathyroidectomy for secondary hyperparathyroidism in patients undergoing PD was 71.2%; (ii) patients who developed HBS after surgery had significantly lower serum calcium, higher ALP, and higher potassium level compared to the non-HBS group; (iii) multivariate logistic regression identified all these three features as independent risk factors after controlling for confounders.

HBS is a common problem observed in ESKD patients who receive parathyroidectomy for refractory SHPT. The incidence of HBS in the present study was 71.2%. Previous literature reported that the incidence of HBS in patients with SHPT varies widely from 10.9% to 87.8% (18, 19, 20, 21, 22, 23, 24, 25). This is mainly due to the lack of well-defined criteria of HBS (26), various protocols for perioperative evaluation of patients, and the different parameters measured in each study (27). For example, HBS was defined in one study as a decrease in serum total calcium to less than 8.4 mg/dL and/or prolonged hypocalcemia for more than four days after parathyroidectomy (24), while in another study it was defined as the requirement of intravenous calcium administration due to clinical symptoms of hypocalcemia and/or a reduction in serum calcium level to less than 8.4 mg/dL during the first 72 h after parathyroidectomy (19). In one recent study, the incidence was reported to be 82.1% when HBS was defined as hypocalcemia with a cutoff value of 8.5 mg/dL that lasted more than 3 days (12), while the incidence reported by Jofré et al. using a more stringent definition of a calcium concentration less than 8 mg/dL was only 20% (25). Thus, there is still an unmet need for a standard regarding the diagnosis and definition of HBS despite the growing interest in research on this topic. The various degrees of SHPT in different patient cohorts across studies, reflected by the serum iPTH levels, might also affect the incidence (12). It is also noteworthy that most previous studies either focused on patients receiving HD or pooled patients on HD and PD into a single study population, while there are few investigations targeting PD patient cohorts (28, 29). The heterogeneity of patients also explains the inconsistency of incidence across studies. Our study adopted the definition of serum corrected calcium lower than 8.4 mg/dL that lasted more than 3 days and only focused on patients receiving PD. The incidence of HBS reported in our study is relatively high but is consistent with previous results from patients with similar characteristics, which may be associated with the high preoperative serum iPTH level (1768.4±1480.3 pg/mL) (29).

Similarly, the lack of standards regarding the definition of diagnosis, the different cutoff values of iPTH, and the different follow-up durations adopted in previous studies have made the actual rates of recurrent SHPT difficult to estimate. The overall recurrence rate in our patient cohorts was 47%, which is higher than the reported recurrence ranging from 10 to 28% in previous studies (15, 21, 30, 31). This could result from the relatively lower threshold iPTH value (200 pg/mL vs 595 pg/mL used by some researchers) we used to detect recurrence. However, our recurrence rate is still on the high side even when compared with the study using the same cutoff value for diagnosis (15, 16). This could result from a relatively higher percentage of patients receiving subtotal parathyroidectomy instead of total parathyroidectomy and the fact that not all patients in our study cohort had received a perioperative parathyroid nuclear scan (Table 3). Due to the heterogeneity of diagnostic standards and patient cohorts, comparing the results from different studies directly becomes challenging. Therefore, it is important to exercise excessive caution and carefully consider these factors when interpreting the data. Additional studies that provide more explicit details about these factors, such as diagnostic standards and patient profiles, are needed to assess this important outcome precisely.

Because the symptoms of hypocalcemia negatively impact patients’ quality of life and could be life-threatening, active identification of risk factors for HBS helps predict the occurrence of HBS and improve preoperative education and patient safety. The present study demonstrated three factors as independent risk factors for postparathyroidectomy hypocalcemia, including preoperative serum calcium, serum ALP, and serum potassium levels.

Our study is consistent with previous research reporting that patients with HBS showed a significantly lower preoperative serum calcium level as compared with patients without HBS (18, 19, 21, 22, 29). Some researchers speculated that it might be because that patients with preoperative hypercalcemia have a higher threshold for HBS (19), although the exact mechanism remains unknown.

We also found that patients who developed HBS had significantly higher preoperative serum ALP levels, which agrees with a handful of previous studies (12, 19, 22, 29). ALP is mainly expressed in bone, liver, kidneys, intestines, and leukocytes and is partly released into the circulation. Bone ALP and liver ALP constitute about 95% of the total ALP activity in human serum (32). Fluctuation of serum ALP levels gives a hint of the alteration of bone-specific ALP in patients with normal liver function and reflects the severity of bone histologic change in patients with renal osteodystrophy (33). It has also been reported that a higher ALP is associated with increased mortality in patients with ESKD receiving maintenance HD or PD (34, 35, 36). Thus, it is plausible that patients with higher preoperative serum ALP levels have a higher bone turnover rate and thus are more susceptible to HBS. We did notice that the ALP concentrations measured in our study, no matter in HBS or non-HBS group (178.2 ± 87.5 vs 115.0 ± 29.6 U/L; mean = 159.7 ± 80.4 U/L), were relatively lower as compared with data from previous work (300- 500U/L)(18, 19, 22, 29). However, as mentioned previously, most studies on HBS were mainly based on observations of HD patients, which is also true for studies that included both HD and PD patients. The numbers of PD patients in previous studies were generally low and might not reflect the pathophysiological characteristics of this distinct cohort. To date, studies that exclusively investigate HBS in PD patients are very limited, but we found one nationwide study evaluating the roles of serum calcium, phosphorus, iPTH and ALP on mortality in 12,116 PD patients, in which the mean ALP was 166.2 ± 120 U/L in patients with iPTH >600 pg/mL, and an ALP exceeding 150 U/L is associated with a higher mortality rate (37). The values they identified, along with the associated pathophysiological significance, are in line with what our study demonstrated. Interestingly, another study focusing on PD patients also found that higher ALP level (>150 U/L) was associated with increased mortality, while a U-shaped association between iPTH concentration and death risk was observed; only patients with iPTH concentrations of less than 200 pg/mL or more than 700 pg/mL had increased mortality (34). Such discrepancy indicates that ALP, albeit having been reported to be positively associated with iPTH in ESKD patients (38), might better reflect bone turnover rate than iPTH does in PD cohorts. This could also explain the lack of significant difference in preoperative iPTH concentrations in HBS vs non-HBS groups. Further studies are required to elucidate whether the disconnection of pathophysiological roles of iPTH and ALP is a phenomenon exclusive to PD patients.

Another intriguing finding of our study is that higher preoperative serum potassium was strongly associated with the occurrence of HBS, which was further confirmed as a novel predictive factor for HBS with multivariate logistic regression analysis. This has never been reported previously, either in studies evaluating HD patients or combined analyses of HD plus PD cohorts. Several studies have established that postoperative hyperkalemia, often accompanied by other electrolyte aberrations including hypocalcemia and hypophosphatemia, is common in ESKD patients receiving parathyroidectomy for SHPT (39, 40). However, the discussions were only limited to postoperative data, and we can barely find any research reporting the association between preoperative potassium level and the development of severe hypocalcemia after surgery. Experience from animal studies suggests that iPTH may affect potassium homeostasis by enhancing calcium entry into cells and increasing calcium content in various tissues, which prevented potassium load translocating from extracellular space to cells and increased serum potassium levels in a chronic renal failure model created with 7/8 nephrectomy in rats (41). It has also been established that SHPT decreases potassium entry inside the cell through increased intracellular calcium, which suppresses the oxidative metabolism and cellular ATP production and reduces the Na^+^/K^+^- ATPase activity (42). These collectively suggest that hyperkalemia observed in the HBS group in our study might indicate that iPTH was more actively functioning in these patients, albeit the iPTH levels did not significantly differ from the non-HBS group. In addition, because our research aimed to focus on PD patients, we also reviewed common causes of hyperkalemia in this distinct cohort in the hope of identifying the characteristics that could provide mechanistic insights for the correlations between hyperkalemia and the development of HBS in PD patients. A prospective study investigating hyperkalemia in 779 serum samples from 33 patients on PD for 1−59 months found that a high-potassium diet, PD noncompliance, increased muscle mass, potassium shifts, and the daytime period without PD might contribute to hyperkalemia (43). Another retrospective single-center study evaluating 319 PD patients reported that blood urea nitrogen and creatinine were significantly associated with hyperkalemia in incident PD patients; because they did not find any significant difference in dialysis adequacy or nutritional indices between serum potassium categories, the authors speculated that the hyperkalemia in these patients might result from the free dietary intake (44). Moreover, hyperkalemia can be a direct cause of metabolic acidosis (45), which is a major precipitating and exacerbating factor of renal osteodystrophy (46). Because we did not routinely collect serum bicarbonate or carbon dioxide levels during postoperative surveillance, data on acid–base status of the patients were incomplete and not analyzable, but metabolic acidosis is a possible explanation for high baseline bone turnover status and proneness to HBS after parathyroidectomy, which corresponds to the results of the two studies investigating hyperkalemia in PD patients we discussed above. Taken as a whole, these studies suggest that hyperkalemia, as a reflection of patients’ adherence to lifestyle and diet modifications, may impact the incidence of HBS, although further studies are warranted to determine the exact mechanisms. Including information such as acid–base status, dietary intakes, and the availability of nutritional and lifestyle recommendations may be a critical approach for future research.

Our study has several limitations. First, it is a retrospective cohort study and thus is subject to flaws associated with this approach, including reporting bias and missing data. Certain important patient characteristics and parameters such as acid–base status could not be analyzed. Other drawbacks include the lack of exploration of the molecular mechanism and the fact that it was a single-center study. However, although there have been abundant studies on the risk factors of HBS in ESKD, studies investigating the topic focusing on PD patients have been scarce. One of the strength points of our study is that we only analyzed PD patients, and the sample number (*n* = 66) allowed for more sufficient and consistent observations as compared with data from sporadic PD patients pooled with HD population in most previous studies on HBS. Also, this is the first study demonstrating that preoperative hyperkalemia may be linked to the development of HBS. In terms of pathophysiology of disease or socioeconomic status, PD patients significantly differ from HD patients, and the information provided in the present study adds value to the preoperative education and postoperative management in this distinct patient cohort.

## Conclusion

Hungry bone syndrome is common (71.2%) after parathyroidectomy for SHPT in patients undergoing PD and should be monitored and appropriately managed. Nevertheless, no significant correlations existed between the development of HBS and patients’ outcomes. In this study, we demonstrated three independent risk factors for postoperative HBS, including lower calcium, higher ALP, and higher potassium in preoperative blood surveys. To the best of our knowledge, our work is not only one of the very limited studies that discussed HBS specifically in PD patients but also the first to report hyperkalemia predictive of HBS. Our results might reflect the hyperfunction of iPTH, high bone turnover, and patients’ dietary factor. Although more studies and mechanistic approaches are needed to validate our results, the data could be helpful to improve the perioperative care in the clinic and provide directions for future research.

## Declaration of interest

The authors declare no conflict of interest.

## Funding

This research was funded by Chang Gung Memorial Hospital, grant number CORPG3M0391 and CORPG3K0195.
